# Antral early gastric neoplasm with severe oral scarring successfully treated by endoscopic submucosal dissection using retroflex approach and a novel, thin therapeutic endoscope

**DOI:** 10.1055/a-2371-0911

**Published:** 2024-08-07

**Authors:** Ryohei Maruoka, Mitsuru Esaki, Yosuke Minoda, Mei Tadokoro, Kazuhiro Haraguchi, Haruei Ogino, Eikichi Ihara

**Affiliations:** 1 Department of Gastroenterology, Harasanshin Hospital, Fukuoka, Japan; 2 Department of Medicine and Bioregulatory Science, Graduate School of Medical Sciences, Kyushu University, Fukuoka, Japan; 3 Department of Metabolism and Gastroenterology, Graduate School of Medical Sciences, Kyushu University, Fukuoka, Japan

Endoscopic submucosal dissection (ESD) for early gastric neoplasms (EGNs) may be associated with challenges. A recently developed endoscope (EG-840TP; Fujifilm Co., Tokyo, Japan), with a thinner outer diameter (7.9 mm) that is equipped with a waterjet function and a large channel (3.2 mm), facilitates easy cleaning and suction without device restrictions during therapeutic procedures 1 2 3 4 . The small radius of curvature enables the procedure to be performed in the retroflex view and in a narrow space ( Fig. 1 , Fig. 2 ). Herein, we describe a case of antral EGN with severe oral scarring following ESD that was successfully treated by ESD using the retroflex approach and the thin therapeutic endoscope ( Video 1 ).

**Fig. 1:**
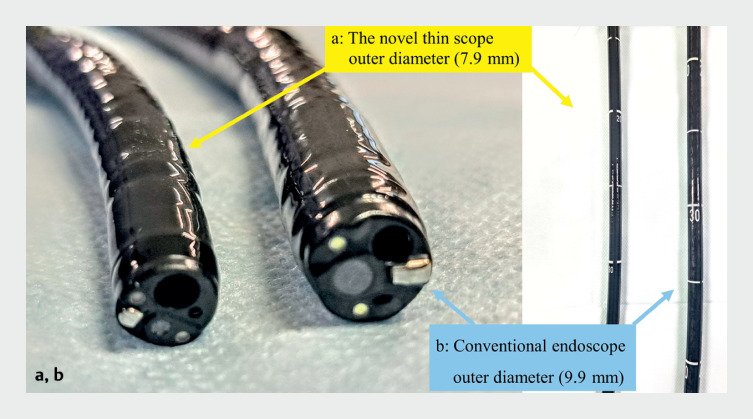
Comparison of the outer diameter. **a** The novel, thin therapeutic endoscope has a thinner outer diameter (7.9 mm) than the conventional therapeutic endoscope. **b** The conventional endoscope has an outer diameter of 9.9 mm.

**Fig. 2:**
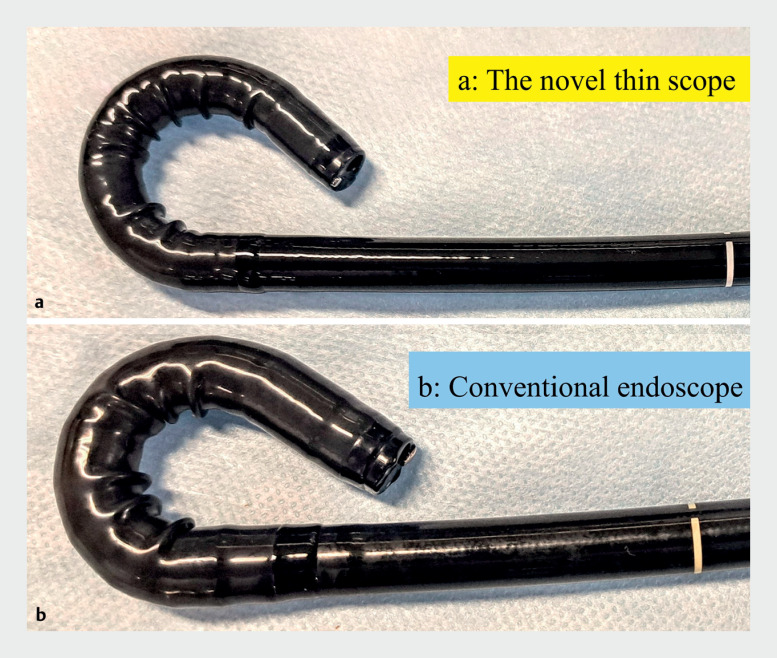
Comparison of scope caliber and bending characteristics. **a** The novel, thin therapeutic endoscope. **b** The conventional endoscope.

Antral early gastric neoplasm with severe oral scarring successfully treated by endoscopic submucosal dissection using the retroflex approach and a novel, thin therapeutic endoscope.Video 1

A 71-year-old man underwent ESD twice for EGNs on the anterior wall of the gastric angle. An elevated 15-mm lesion was identified on the anterior wall of the gastric antrum, just on the anal side of the post-ESD scar ( Fig. 3 ). Although ESD using a conventional therapeutic endoscope enables endoscopic procedures to be performed with the forward approach, the retroflex approach cannot be used because of the narrow space of the antrum. With the forward approach, marking dots can be placed around the lesions using an electrosurgical knife. However, mucosal elevation was not achievable on the oral side of the lesion because of severe post-ESD scarring. Switching to the thin therapeutic endoscope enabled the retroflex approach to be used with a sufficient distance for the endoscopic procedure, even in the narrow space ( Fig. 4 ). Mucosal injection was followed by mucosal incision and submucosal dissection, which were all performed carefully at the fibrotic site, proceeding from the anal to the oral side ( Fig. 5 ). En bloc resection was successfully performed without complications.

**Fig. 3:**
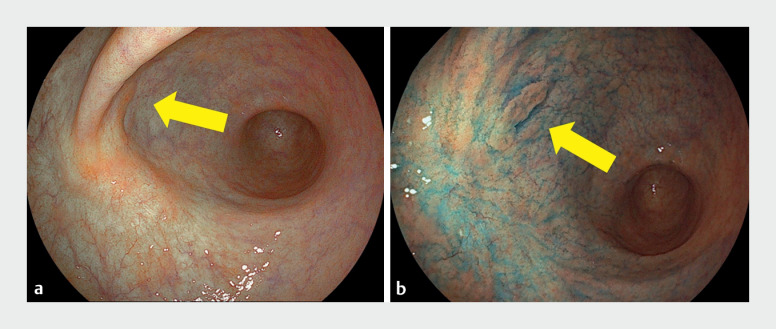
White-light images of the lesion and the scar (yellow arrows) following endoscopic submucosal dissection (ESD). **a** White-light image from the oral perspective. **b** The lesion and post-ESD scar after the application of indigo carmine dye.

**Fig. 4:**
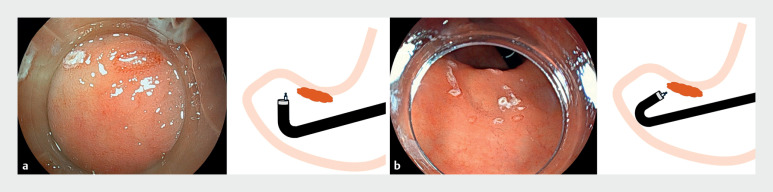
Endoscopy images using the retroflex approach. **a** Conventional therapeutic endoscope. **b** Novel, thin therapeutic endoscope.

**Fig. 5:**
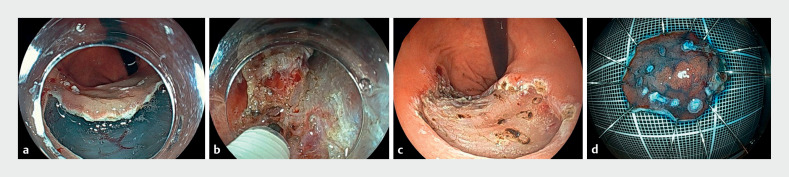
Steps of the endoscopic submucosal dissection procedure. **a** Using the retroflexion approach, mucosal incision and submucosal dissection are performed from the anal to the oral side. **b** Mucosal incision and submucosal dissection are performed carefully at the fibrotic site. **c** The artificial ulcer after endoscopic submucosal dissection is shown. **d** Resected specimen showing that en bloc resection was achieved.

The novel, thin therapeutic endoscope offers a viable option for ESD in challenging situations.

Endoscopy_UCTN_Code_TTT_1AO_2AG_3AD

## Notes

Endoscopy E-Videos https://eref.thieme.de/e-videos
*E-Videos* is an open access online section of the journal *Endoscopy* , reporting on interesting cases and new techniques in gastroenterological endoscopy. All papers include a high-quality video and are published with a Creative Commons CC-BY license. Endoscopy E-Videos qualify for HINARI discounts and waivers and eligibility is automatically checked during the submission process. We grant 100% waivers to articles whose corresponding authors are based in Group A countries and 50% waivers to those who are based in Group B countries as classified by Research4Life (see: https://www.research4life.org/access/eligibility/ ). This section has its own submission website at https://mc.manuscriptcentral.com/e-videos .
